# Nrf2 Pathway Ameliorates Bladder Dysfunction in Cyclophosphamide-Induced Cystitis via Suppression of Oxidative Stress

**DOI:** 10.1155/2021/4009308

**Published:** 2021-06-30

**Authors:** Bin Ni, Zhengsen Chen, Le Shu, Yunpeng Shao, Yi Huang, Nebiyu Elias Tamrat, Zhongqing Wei, Baixin Shen

**Affiliations:** Department of Urology, The Second Affiliated Hospital of Nanjing Medical University, Nanjing 210000, China

## Abstract

**Objective:**

To investigate the protective effect and molecular mechanism of nuclear factor E2-related factor 2 (Nrf2) pathway in interstitial cystitis (IC).

**Methods:**

We established a mouse model of IC by cyclophosphamide (CYP) in wild-type mice and Nrf2 gene knockout mice. We examined the histological and functional alterations, the changes of oxidative stress markers, and the expression of the antioxidant genes downstream of Nrf2 pathway.

**Results:**

After CYP administration, the mice showed urinary frequency and urgency, pain sensitization, decreased contractility, bladder edema, and oxidative stress disorder. Notably, the Nrf2^−/−^ CYP mice had more severe symptoms. The mRNA and protein levels of antioxidant genes downstream of Nrf2 pathway were significantly upregulated in the Nrf2^+/+^ CYP mice, while there were no significant changes in the Nrf2^−/−^ CYP mice.

**Conclusion:**

Nrf2 pathway protects bladder injury and ameliorates bladder dysfunction in IC, possibly by upregulating antioxidant genes and inhibiting oxidative stress.

## 1. Introduction

Interstitial cystitis (IC), also known as bladder painful syndrome (BPS), is a chronic inflammatory bladder disease that clinically manifests as pain or pelvic discomfort related to bladder filling, accompanied by lower urinary tract symptoms such as urinary urgency and frequency [1]. There is wide variation in reported incidence and prevalence of IC depending on the criteria used for diagnosis, but approximately 90% of the patients are women [2]. At present, the therapeutic alternatives are still limited and unsatisfactory. Therefore, further research is needed to clarify the related mechanism of IC.

Recently, increasing studies have found that oxidative stress is closely associated with pathological mechanisms of various diseases [3]. Reactive oxygen species (ROS) produced by oxidative stress may contribute to bladder dysfunction [4]. Previous research has found that diverse antioxidants could suppress oxidative stress induced by IC [5, 6]. The nuclear factor E2-related factor 2 (Nrf2) pathway is a transcription factor involved in regulating the cellular antioxidative responses by promoting the expression of ant genes through binding to antioxidant response element (ARE, 5′-TGACXXXGC-3′) [7]. Under homeostatic conditions, two molecules of keap1 are bound to Nrf2 and isolate Nrf2 in the cytoplasm [8]. In response to oxidative stress, Keap1 inactivation promotes the dissociation of Nrf2 from Keap1 and translocation into the nucleus. The Nrf2-sMaf complex binds, in a sequence-specific manner, to the ARE in the promoter region of Nrf2 target genes and then initiates the expression of a series of antioxidant genes, such as heme oxygenase-1 (HO-1), NAD (P)H : quinone oxidoreductase 1 (NQO1), glutathione reductase (GR), and superoxide dismutase (SOD) [9]. However, the specific molecular mechanism of Nrf2 in IC is still unclear and the functional evaluation of IC animal models remains vague.

In this study, wild-type and Nrf2 knockout mice were obtained to establish the IC model by cyclophosphamide (CYP). Moreover, our study is the first to establish an objective functional evaluation system for the study of the bladder function and behavior in IC animal models, according to the typical clinical manifestation of IC. Micturition behavior, pain hypersensitivity, and urodynamics were performed to assess the IC animal models. In summary, the protective effect and regulation mechanism of the Nrf2 pathway in IC were explored, in order to provide new options for the clinical treatment of IC.

## 2. Materials and Methods

### 2.1. Animals

Wild-type C57BL/6J mice were obtained from the Animal Center of Nanjing Medical University and the Nrf2 knockout mice were purchased from the Model Animal Research Center of Nanjing University. All mice were housed five per cage in a room under a 12 h light/dark cycle with free access to food and water under the condition of 20–26°C with 40–60% relative humidity. The animals in this study were sacrificed by a physical method of euthanasia under anesthetized with isoflurane. All experimental procedures were approved by the Animal Ethical and Welfare Committee of Nanjing Medical University (IACUC-1904032).

A total of 80 mice were randomly divided into four groups (*n* = 20): Nrf2^+/+^ control group, Nrf2^−/−^ control group, Nrf2^+/+^ CYP group, and Nrf2^−/−^ CYP group. The control groups received saline treatment, and the CYP groups received a single intraperitoneal injection of cyclophosphamide (CYP, 150 mg/kg) [10]. The mice were sacrificed 24 hours after administration. Mice in the same group received homogeneous treatment.

### 2.2. Void Spot Assays (VSA)

**Figure 1:**
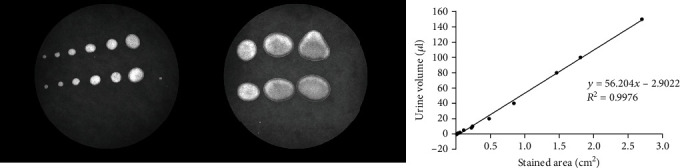
Urine was collected to construct the standard curve and the urine was pipetted onto filter paper in different volumes (1, 2, 4, 10, 20, 40, 80, 100, 150 *μ*l). The formula *Y* (volume) = 56.204∗*X* (stained area) − 2.9022 (*R*^2^ = 0.9976) was used to calculate individual void on the filter paper.

Urinary frequency and mean voided volume were studied using the void spot assay [11]. We performed the VSA experiments 24 hours after CYP administration in a circle metabolic cage (Yuyan, Shanghai). Individual mice were gently removed into the metabolic cage with circular filter paper (Whatman No.1) taped to the bottom without a grid. The mice were provided with standard food and water for the duration of the assay. The micturition cages were kept in a quiet area for 2 hours. At the end of 2 hours, the mice were returned to normal housing, and the filter paper was recovered and imaged using an imaging system. The images were analyzed by the Fiji version of the ImageJ software. Urine volumes were determined using an area-to-volume standard curve as shown in Figure 1. We performed the VSA experiments at approximately the same time every afternoon between 9 AM and 4 PM.

### 2.3. Pelvic Nociceptive Response Using von Frey Filaments

Mice were placed individually in Plexiglas cubicles on a wire grid on a raised platform (Yuyan, Shanghai). Mice were allowed to acclimate to the environment for a minimum of 30 minutes before testing. Filaments were applied vertically to the pelvic area close to the bladder. The 50% withdrawal threshold was determined using the up-down method of Dixon (1980) [12]. We chose 0.008 g, 0.02 g, 0.04 g, 0.07 g, 0.16 g, 0.4 g, 1 g, 2 g (North coast, USA) as a series and the testing was initiated with the 0.07 g hair, in the middle of the series. Withdrawal or retraction of the lower abdominal/pelvic area from the filament stimulation was considered a positive response. In the absence of a withdrawal response to the initially selected filament, a stronger stimulus was chosen; in the event of withdrawal, a weaker stimulus was chosen. According to Dixon, once the two responses straddle the threshold, four additional responses are required.

### 2.4. Urodynamic Measurements

Urodynamic measurements were performed 24 hours after CYP administration in mice as previously described [13]. The mice were anesthetized with isoflurane (2%). Before the start of the recording, the air in the system was emptied and the catheter was connected via a T-tube to a pressure transducer (Taimeng, Chengdu) and microinjection pump (Silugao, Beijing). Normal saline was infused at room temperature into the bladder at a rate of 3 ml/h to elicit repetitive bladder contractions. Continuous urodynamic curves were digitized and recorded using a multichannel signal processing system (Taimeng, Chengdu) for at least 30 minutes. The micturition pressure and intercontractile interval were analyzed, which represent the bladder contractility and urinary frequency. We performed the urodynamic measurements at approximately the same time every afternoon between 9 AM and 4 PM.

### 2.5. Bladder Histology

Mice bladders were obtained and processed with 4% paraformaldehyde fixation, paraffin embedment, 5 *μ*m section preparation, dewaxing, hematoxylin and eosin (HE) staining, dehydration, and photography, according to the standard steps.

### 2.6. Oxidative Stress Markers Determination

This study uses the method of Liu et al., and the method description partly reproduces their wording [14]. The contents of MDA, SOD, and GSH-Px in the bladder tissues were determined by spectrophotometry according to the manufacturer's protocols (Jiancheng, Nanjing). Briefly, the MDA level was detected using the thiobarbituric acid (TAB) method, and the maximum absorbance was read at 532 nm. The activity of SOD was based on the combination of xanthine and xanthine oxidase, and the maximum absorbance was read at 450 nm. The activity of GSH-Px was measured using the enzyme-catalyzed reaction product, and the maximum absorbance was read at 412 nm.

### 2.7. Western Blot

Mice bladders were lysed in RIPA lysis buffer (Beyotime, Shanghai) to extract total protein. Protein concentrations were measured with BCA. Then, 30-50 *μ*g protein aliquots were separated using 10% sodium dodecyl sulfate polyacrylamide gel electrophoresis (SDS-PAGE) gels and transferred to polyvinylidene fluoride (PVDF) membranes. After being blocked with 5% skim milk dissolved in TBST at room temperature for 2 h, the membranes were incubated overnight at 4°C with various primary antibodies: HO-1 (1 : 1000, Affinity), NQO1 (1 : 1000, Affinity), and *β*-actin (1 : 5000, Proteintech). Following incubation with horseradish peroxidase-conjugated secondary antibodies (1 : 5000, Proteintech), antibody–antigen complexes were detected using an ECL substrate and visualized with an imaging system.

### 2.8. qRT-PCR

RNAs in tissues were extracted using TRIzol (Invitrogen, USA). The extracted RNA was quantified and stored at -80°C. Reverse transcription experiments were performed according to the instructions of the PrimeScript™ RT reagent Kit with gDNA Eraser (Takara) on GeneAmp PCR System 9700 (Applied Biosystems, USA). Quantitative real-time PCR (qRT-PCR) was performed by TB Green® Premix Ex Taq™ II (Takara) on the Applied Biosystems StepOnePlus Real-Time PCR system (Applied Biosystems, USA). The PCR was performed as follows: incubation at 95°C for 3 min followed by 35 cycles for 30 s at 94°C, 30 s at 57°C, and 30 s at 72°C. The relative expression of mRNA was evaluated using the 2^-*ΔΔ*Ct^ method. *β*-Actin was used as a reference housekeeper gene [15]. Primer sequences were list in Table 1.

### 2.9. Statistical Analysis

All data are shown as mean ± SD. GraphPad Prism 8 was used to evaluate data. Student's *t*-test was used to analyze the differences between the groups. A value of *P* < 0.05 was considered statistically significant.

## 3. Result

### 3.1. Nrf2 Ameliorated Bladder Dysfunction in CYP-Induced Cystitis

**Figure 2:**
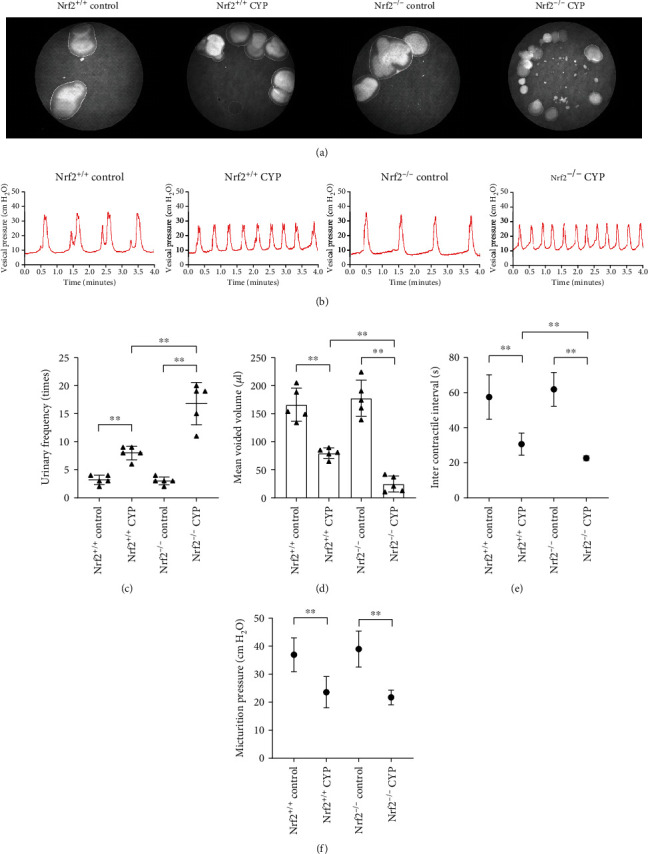
Nrf2 ameliorated bladder dysfunction in CYP-induced cystitis. (a) Sample VSA blots from four groups. (b) Urodynamic curves of four groups. Comparison of urinary frequency (c), mean voided volume (d), micturition pressure (e), and inter contractile interval (f) in four groups. ^∗^*P* < 0.05, ^∗∗^*P* < 0.01. Data are presented as mean ± SD.

As shown, there was no difference between the control groups (*P* > 0.05). After CYP administration, the mice showed urinary frequency and urgency (Figure 2(a)). We found the number of micturition increased and mean voided volume decreased compared with the corresponding control mice. Compared with Nrf2^+/+^ CYP mice, Nrf2^−/−^ CYP mice had more severe performance (Figures 2(c) and 2(d)).

Furthermore, the urodynamics of the mice were examined under anesthesia, and the parameters of cystometry were measured according to the urodynamic curve (Figure 2(b)). The inter contractile interval was significantly shorter in CYP mice than in control mice. However, the intercontractile interval in Nrf2^−/−^ CYP mice significantly decreased compared with that in Nrf2^+/+^ CYP mice (Figure 2(e)). Micturition pressure significantly decreased in CYP mice; however, no significant difference was observed in micturition pressure between Nrf2^+/+^ CYP mice and Nrf2^−/−^ CYP mice (Figure 2(f)). The results showed that Nrf2 ameliorated bladder dysfunction in CYP-induced cystitis.

### 3.2. Nrf2 Alleviated the Pelvic Hypersensitivity in CYP-Induced Cystitis

**Figure 3:**
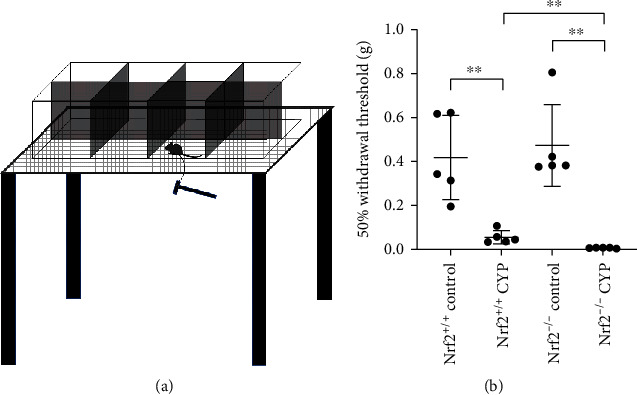
Nrf2 alleviated the pelvic hypersensitivity in CYP-induced cystitis. (a) Schematic diagram of determination of withdrawal threshold using von Frey filaments. (b) 50% withdrawal threshold of four groups. ^∗^*P* < 0.05, ^∗∗^*P* < 0.01. Data are presented as mean ± SD.

As a typical clinical manifestation of IC, allodynia in the suprapubic area was determined using von Frey filaments (Figure 3(a)). The pelvic hypersensitivity was represented by 50% withdrawal threshold, which significantly decreased in CYP mice than in control mice. Importantly, Nrf2^−/−^ CYP mice showed lower withdrawal threshold compared with Nrf2^+/+^ CYP mice (Figure 3(b)).

### 3.3. Nrf2 Rescued the Histological Changes in CYP-Induced Cystitis

**Figure 4:**
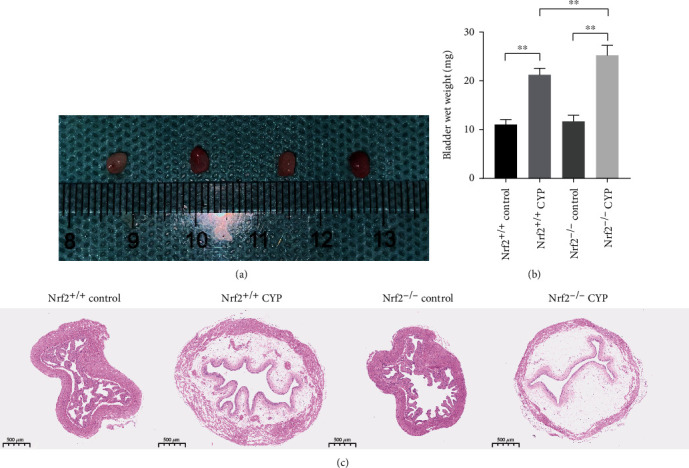
Nrf2 rescued the histological changes in CYP-induced cystitis. (a) Representative images of bladder from four groups. (b) Comparison of bladder weight in four groups. (c) Representative histological bladder sections from four groups (magnification: 20x). ^∗^*P* < 0.05, ^∗∗^*P* < 0.01. Data are presented as mean ± SD.

CYP administration caused bladder injury, as manifested by the appearance of bladder edema and congestion (Figure 4(a)). The bladder weight significantly increased in CYP mice and Nrf2^−/−^ CYP mice had higher wet weight than Nrf2^+/+^ CYP mice (Figure 4(b)). HE staining revealed that CYP administration resulted in bladder edema and structural destruction. However, Nrf2^−/−^ CYP mice showed more severe damage in histology (Figure 4(c)). The findings indicated that Nrf2 rescued the histological changes in CYP-induced cystitis.

### 3.4. Nrf2 Attenuated Oxidative Stress in CYP-Induced Cystitis

**Figure 5:**
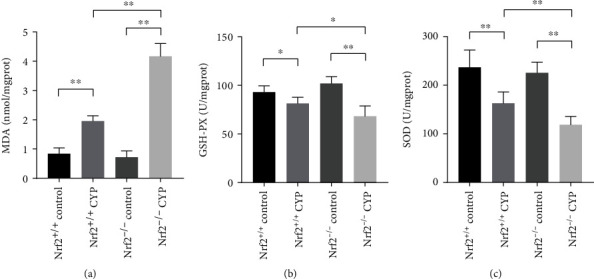
Nrf2 attenuated oxidative stress in CYP-induced cystitis. (a) MDA level in the bladder of the four groups. (b) The activities of GSH-Px in the bladder of the four groups. (c) The activities of SOD in the bladder of the four groups. ^∗^*P* < 0.05, ^∗∗^*P* < 0.01. Data are presented as mean ± SD.

MDA, SOD, and GSH-Px were measured to evaluate the level of oxidative stress. Our study showed that the content of MDA in bladder was significantly increased in CYP mice compared with the corresponding control mice. Furthermore, the level of MDA was higher in Nrf2^−/−^ CYP mice than in Nrf2^+/+^ CYP mice (Figure 5(a)). The activities of SOD and GSH-Px significantly decreased in CYP mice compared with the corresponding control mice. However, the activities of SOD and GSH-Px was lower in Nrf2^−/−^ CYP mice than in Nrf2^+/+^ CYP mice (Figures 5(b) and 5(c)). The results indicated that Nrf2 attenuated oxidative stress in CYP-induced cystitis.

### 3.5. Nrf2 Might Protect against Bladder Injury by Activating Its Downstream Antioxidant Genes

**Figure 6:**
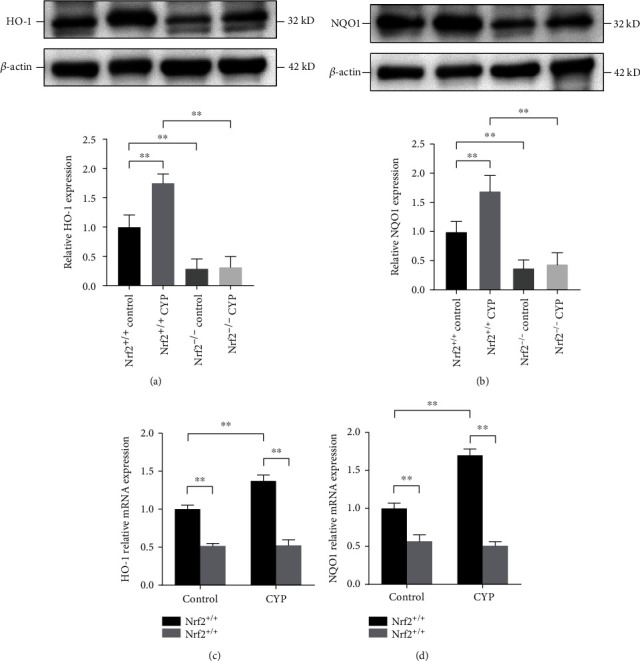
Nrf2 might protect bladder injury by activating its downstream antioxidant genes. (a) The protein expression of HO-1 in the bladder of the four groups. (b) The protein expression of NQO1 in the bladder of the four groups. Relative mRNA expression levels of HO-1 (c) and NQO1 (d) in the bladder of the four groups. ^∗^*P* < 0.05, ^∗∗^*P* < 0.01. Data are presented as mean ± SD.

In order to explore the mechanism of Nrf2 in CYP-induced cystitis, we investigated the expression of its downstream antioxidant genes. We found that both the mRNA and protein levels of HO-1 and NQO1 were upregulated in Nrf2^+/+^ CYP mice compared with Nrf2^+/+^ control mice. As expected, there was no obvious expression in Nrf2^−/−^ mice (Figures 3(a)–3(d)). The results demonstrated that Nrf2 protected mice against bladder injury, possibly through activating its downstream antioxidant genes. The Figure 6 is shown below.

## 4. Discussion

The etiology of IC is unknown and there are few effective clinical treatments for IC. Therefore, exploration of more alternative treatments for IC is necessary. Nrf2 pathway has been proven to inhibit oxidative stress damage in cardiac myocyte damage [16], lung injury [17, 18], and liver damage [19, 20]. Increasing studies have shown that oxidative stress participates in IC [21]. Moreover, a study demonstrates that the serum antioxidant capacity in IC patients is lower than that in controls [22]. As the crucial regulator of antioxidant defense system, Nrf2 pathway may play a critical role in the occurrence and development of IC. According to relevant studies, CYP-induced cystitis is the most stable, reliable, and widely used mode for the study of IC [23]. The typical clinical manifestations of IC can be induced after a single high-dose injection, such as pain-related behavior, bladder edema, urinary frequency, and urgency [24]. According to the symptoms above, our study is the first to establish an objective functional evaluation system for the study of the function and behavior in IC animal models, which provides a reference method for the related research in the future. However, animal models for the study of IC need further optimization.

It has been reported that Nrf2 could ameliorate bladder dysfunction through suppressing oxidative stress [25, 26]. In this study, we performed some interesting experiments, such as void spot assay, determination of pain threshold using von Frey filaments, and urodynamic examination. Previous researches just adapted some of them to evaluate bladder hyperreflexia induced by CYP [27]. As reported, we observed the urination frequency and mean voided volume. The 50% withdrawal threshold was determined using the up-down method of Dixon. At the same time, the micturition pressure and the micturition interval are measured in the urodynamic examination. We found that the CYP mice showed varying degrees of bladder dysfunction and behavioral changes, but Nrf2^−/−^ CYP mice had more severe symptoms. The results indirectly indicated that Nrf2 might play a vital role in ameliorating IC-induced bladder dysfunction.

In addition, we performed HE staining on the mice bladder. CYP administration could result in extensive cystitis, bladder edema and structural destruction [28]. Notably, Nrf2^−/−^ CYP mice had more severe symptoms. Our study demonstrated that Nrf2 could protect mice against IC-induced morphological damage. Furthermore, we measured the oxidative stress markers in mice bladder. We found that knockout of Nrf2 caused higher sensitivity to oxidative stress in CYP mice. Nrf2^−/−^ CYP mice showed higher level of MDA and decreased activities of SOD and GSH-Px compared with Nrf2^+/+^ CYP mice. The results indicated that Nrf2 might alleviate oxidative stress in IC.

Finally, we further studied the potential mechanism of Nrf2 pathway in IC. The expression of the antioxidant enzymes downstream of Nrf2 pathway, including HO-1 and NQO1, was determined. HO-1 is an antioxidant enzyme encoded by the HMOX1 gene, which can catalyze the decomposition of heme and exert antioxidant effects through the decomposition products [29]. NQO1 is a ubiquitous cytosolic phase II biotransformation enzyme whose primary physiological role is catalysis of two-electron reduction of quinones and thereby their detoxification. Knockout of NQO1 gene in mice led to increased reactive oxygen species, alterations in factors regulating energy metabolism and adhesion, and loss of mitochondrial structures including cristae structures [30]. We found that after knocking out Nrf2, the endogenous HO-1 and NQO1 levels were significantly lower than those in normal mice. Importantly, their activities could not be upregulated after CYP administration, which suggested that Nrf2 is an essential translation factor for regulating the expression of antioxidant genes. It has been reported that the expression of Nrf2 and its downstream antioxidant genes decreased after other drug administration [31, 32]. Difference of drugs and the duration of intervention may contribute to the discrepancy. Some poison administration may directly or indirectly cause DNA damage, thereby affecting gene transcription regulation. Moreover, in response to oxidative stress injury, Nrf2 translocates into the nucleus and then activates its downstream antioxidant genes, which may show a curvilinear change. Our previous study showed that the expression of Nrf2 and its downstream antioxidant genes gradually increased after CYP administration. However, the expression levels reached their peak at 24 hours and gradually decreased subsequently, which is consistent with previous research [33, 34]. The results indicated that Nrf2 might protect mice against bladder dysfunction and oxidative stress by activating its downstream antioxidant genes in IC. Based on current findings, it is significant to explore Nrf2-related pharmaceutical treatments for IC.

In summary, our study is the first to adopt Nrf2 knockout mice in the study of IC and establish an objective functional evaluation system for IC animal models. We found that Nrf2 pathway protected mice against CYP-induced cystitis, possibly by activating the expression of antioxidant genes to inhibit oxidative stress and ameliorate bladder dysfunction. These findings may provide strong evidence for the Nrf2 pathway as a new target for clinical treatment of IC.

## Figures and Tables

**Table 1:** Prime sequence.

Gene	GeneBank	Primer sequence (5′-3′)	Product size (bp)
Nrf2	NM_010902	F: TTCCTCTGCTGCCATTAGTCAGTC	215
		R: GCTCTTCCATTTCCGAGTCACTG	
HO-1	NM_010442	F: ATCGTGCTCGCATGAACACT	339
		R: CCAACACTGCATTTACATGGC	
NQO1	NM_008706	F: ACTCGGAGAACTTTCAGTACC	492
		R: TTGGAGCAAAGTAGAGTGGT	
*β*-Actin	NM_007393	F: AGTGTGACGTTGACATCCGTA	150
		R: GCCAGAGCAGTAATCTCCTTCT	

## Data Availability

All of data were presented in the main paper. The data that support the findings of this study are available on request from the corresponding author. All authors take responsibility for the integrity of the data and the accuracy of the data analysis.
